# Measuring the reliability of proxy respondents in behavioural assessments: an open question

**DOI:** 10.1007/s40520-023-02501-z

**Published:** 2023-08-04

**Authors:** Antonella Lopez, Luigi Tinella, Alessandro Caffò, Andrea Bosco

**Affiliations:** 1 Faculty of Law, Giustino Fortunato University, Via Delcogliano, 12, Benevento, Italy; 2 Department of Educational Sciences, Psychology, Communication, University of Bari, Via Crisanzio 42, 70122 Bari, Italy

**Keywords:** Proxy informant, Observers, Raters, Ageing, Psychometrics, Measurement reliability

## Abstract

**Background:**

In behavioural assessment, information can be gathered from internally referenced self-reports or from proxy informants.

**Aims:**

This study aimed to fine-tune a brief but reliable method for evaluating the proxy accuracy in cases where responses obtained from adult and older adults’ patient cannot be considered reliable.

**Methods:**

We generated a set of items reflecting both overt and covert behaviours related to the basic instrumental activities of daily living. The psychometric properties of the content, factorial, and criterium validity of these items were then checked. The Proxy Reliability Questionnaire—ProRe was created. We tested the frequency of “I don’t know” responses as a measure of proxy reliability in a sample of healthy older adults and their proxies, and in a second sample of proxy respondents who answered questions about their parents.

**Results:**

As expected, response precision was lower for items characterizing covert behaviours; items about covert compared to overt behaviours generated more “I don’t know” answers. Proxies provided less “I don’t know” responses when evaluating the parent, they claimed they knew better. Moreover, we tried to validate our approach using response confidence. Encouragingly, these results also showed differences in the expected direction in confidence between overt and covert behaviours.

**Conclusions:**

The present study encourages clinicians/researchers to how well the proxy the patient know each other, the tendency of proxies to exhibit, for example, response bias when responding to questions about patients’ covert behaviours, and more importantly, the reliability of informants in providing a clinical assessment of neurocognitive diseases associated with aging.

**Supplementary Information:**

The online version contains supplementary material available at 10.1007/s40520-023-02501-z.

## Introduction

Behaviours are an assembly of adaptive responses that a body equipped with a nervous system performs as a response to environmental stimuli which are also objectively observable [1]. This definition of behaviour emphasizes a person’s experience and socio-cultural context, suggesting that mental states, personality structure, physiology, neurology, genetic expression [2], and behavioural repertoires change.

Behavioural assessment is a powerful and well-established psychological paradigm [3] that can be divided into five types: (a) direct assessment, the study of behaviour as it changes during a given situation, also known as ‘Situational behavioural assessment’. However, it could not accurately capture an individual’s typical behaviour if the method is not standardized; (b) analog assessment, a type of behavioural assessment that studies behavioural change under simulated or made-up situations. It could be limited by the validity of the simulated situation, due to inconsistent data coming from different levels of the definition of the relevant behaviours; (c) idiographic assessment that describes the behavioural characteristics of the individual concerned. It could be affected by bias due to narrow definitions of behaviour that may lead to less consistency in observing behaviour; (d) contextual assessment in which the stimuli in the environment that cause the change in behaviour are in focus. The contextual dependence could influence the results, limiting the generalizability of the same evidence; finally, (e) indirect assessment in which the behaviour is not observed but inferred through retrospective analysis. Indirect assessment is the one that is most frequently adopted for its practicality and cost-effectiveness, at least in the initial stages of clinical evaluation. It is an excellent way to gain the understanding of individual characteristics but is susceptible to reporting bias, providing impressions and opinions and not hard evidence; indeed, the major challenge for this method is accuracy: it depends on grasping the significance of the context in which the behaviour was performed, the strength of episodic memory traces, and the respondents’ motivation and integrity in giving the response [4].

### The role of external informants

During an assessment, if patients cannot provide accurate self-reports, it may be important/necessary to make recourse to external informants or proxies. Proxy reports can alternatively be used as a complementary evaluation to improve the accuracy of screening [5]. A proxy is an individual who provides reports on behalf of, or about, a patient, beneficiaries, or nursing home residents. In some cases, they substitute individuals who are unable to reliably self-report their outcomes, for reasons due to cognitive or linguistic impairment that constrains comprehension of items, self-awareness or self-expression, or symptom burden and clinical deterioration in terminal illness. Some aspects of patient health cannot be recorded using behavioural observation scales or performance-based measurement, but through self-report, such as for symptom experience, emotional well-being, and quality of life, in various stages of life. Representative and central examples of proxy reports have been designed specifically for infants and toddlers [6, 7] as well as for people with dementia [8], brain injuries [9], chronic mental illness [10, 11], severe physical illness [12], or those at risk of malingering [13]. In all these cases, patients may not be able to articulate accurately their experience (e.g. young children, people with severe mental illness) or they may be too sick to answer (e.g. end-stage disease), leading to invalid or missing data [14]. In turn, proxy reports could lead the clinician to collect reliable information and increases representativeness in studies [15]. Moreover, proxy ratings represent a way to examine the patient premorbid ability [16] and permit longer follow-up periods, as data collection is not dependent on patient’s capacity to answer [17].

Proxies are essential for minimizing selection bias and preserving external validity [18]. The clinician expects that a proxy’s report can truly integrate/substitute for the patient’s report. Generally, a proxy is a family member, caretaker, or the care staff member who is the closest to the person under evaluation or someone who has significant interactions with the patient. Proxy ratings improve response rates, leaving the costs of data collection practically unchanged [19]. Proxies can help in longitudinal studies where the patient’s condition gets worse over time [16], since data collection is no longer dependent on the patient’s capacity to respond [20]. Moreover, the schooling and the language proficiency of the patient do not seem to affect proxy reports [21].

Some issues may be encountered with proxy data collection. Generally speaking, a critical difficulty with the collection of proxy data is the low level of universality, which makes it more difficult to achieve standardization: different proxy questionnaires must be developed for the different stages of life [22, 23], and, to the best of our knowledge, no proxy questionnaire can be employed across diverse special populations or to assess different functions. However, despite such difficulties, proxy reports are useful for detecting and treating functional impairment and pain in a timely manner [24, 25], for assessing symptoms and the patient’s sense of self-worth, and several other aspects of the health-care situation which patients may not be able to accurately self-report themselves [26–28].

### Observing overt and covert behaviours

A proxy’s ability to assess target behaviours may vary considerably. Overt behaviours [29] (e.g. public behaviours) are manifest and accessible to external observers, while covert behaviours may be less so. Overt behaviour is a type of external behaviour, which can be observed through physical actions (e.g. eating food, driving a car, falling) and can include verbal behaviours, and facial or bodily gestures. Unlike overt behaviour, covert behaviour is not detectable and consists essentially in mental processes. Covert behaviours include thinking, reasoning, retrieving memories, processing information for decision-making, and cognition [30]. Covert behaviours can be considered private behaviours that are unavailable to other persons [31, 32]. Private behaviours always have an audience of one (the subject), while public events have an audience greater than one [33]. Information on covert behaviours is usually collected from internally referenced self-reports since one of the most direct options for understanding psychological processes is to query the first-person perspective [34]. However, in some cases, such as when people are sick, impaired, unavailable, deceased or because another point of view is wanted, responses from a proxy become relevant [35]. In such case sometimes, the distinction between overt and covert behaviours may be blurred, with the proxy providing opinions that are not based accurate information [36] due to mental bias, a motivation to deceive, or to conform with perceived social desirability [37]. For example, especially in the context of behavioural assessment of child and older adults, proxies characterized by depressive or pessimistic may overestimate the patient conditions. Similarly, proxies with the role of caregivers may overstate the incapacities of the person he/she cares for because of the dependent patterns in their relationship [20]. Moreover, if proxy is a caregiver the Fabricated or Induced Illness by Carers may occur, previously known as the Munchausen Syndrome by Proxy, where caregivers intentionally deceive health practitioners about the person symptoms by subjecting him/her to unnecessary and often painful medical procedures [38]. Finally, socially desirable responding and a context dependent bias to respond in certain way, is due to perceived benefits or access to benefits.

### The patient–proxy relationship

The nature of the patient–proxy relationship can influence proxy reports [39, 40]. The type of relationship is formal or informal (e.g. spouse, child, publicly or privately paid home-care professionals), although demographic characteristics of proxies (age, gender, education, and employment status) may play an important role on information reported [20].

For instance, the proxy’s knowledge of the patient can be measured by the amount of time they share with patients, the frequency and intensity of such contact, as well as the quality of their relationship and communication. Usually, the better the proxy’s knowledge and understanding of a patient, the more accurate their evaluation, because a more intimate the proxy/patient relationship, gives proxies more precise, repeated, episodic information regarding the patient’s disease experiences and suffering [41, 42]. It seems that the key issue to consider is the closeness to the patient and the frequency of the interaction with him/her [43]. According to this view, Cummins [44] found substantial differences between family carers and formal carers in a nursing home setting, concerning how often they visit the patient and the accuracy of evaluations.

On the other hand, there are some weaknesses that can affect the quality of information provided by proxy informants. In some cases, they tend to be inaccurate, to emphasize negative information [45], or to respond in what they perceive to be socially desirable with respect to perceived benefits or access to services. In other cases, proxy informants can perceive the patient to be vulnerable and hence in need of overprotection [46]. These psychological–emotional–perceptual mechanisms are relatively common among parents of children/adolescents with chronic conditions, reflecting a level of parental involvement that is excessive, taking into account the child’s actual abilities [47, 48]. Proxy respondents can both over- and underestimate morbidity and disability [40, 49–54]. If proxies are unable to accurately report a patient’s perspective, this can produce misleading information [55]. More specifically, considering clinician-reported outcomes, several studies have reported difficulties of formal caregiver in reporting information (e.g. response precision, different schema, [56]) related to less observable domains (e.g. emotional function) compared to more observable domains of health (e.g. physical function) [57, 58].

Close family members or proxy professionals tend to be capable proxies, although proxy reports may be influenced by caregiving burden, especially for those who provide care to older adults or to people with chronic diseases/health condition. Proxy ratings of disability increased with caregiving hours and higher perceived burden, and proxies reporting more burden tended to report more patient impairment [20, 59]. Evidence showed that caregiver burden may result in misleading representation of the older person’s functional status, specifically in regard to instrumental activities of daily living (IADL) items [60].

### The reliability of proxy reports

Given everything said so far, it is important to assess the reliability of proxy reports, provided that such evaluations are affordable in terms of human resources and time constraints. Moreover, ensuring the reliability of proxy reports avoids the recommendation that they be used only as supplements, rather than replacements, for self-reports [26]. Proxy reliability is usually measured in terms of response precision and response bias [61]. Response precision refers to the degree to which the proxy’s response agrees with the patient’s response. Response bias is systematic over- or underreporting by the rater in comparison with the patient’s report. Both response precision and bias depend on the nature of the construct being assessed, the characteristics of the patient and the proxy, as well as on the characteristics of the items assessed [13, 62, 63]. Generally, the proxy completes an adapted version of the set of questions patients have addressed and the proxy is asked to answer from the patient’s point of view. Although studies have used a wide range of data collection methods and instruments, most have focused on patients’ health outcomes and quality of life [64].

Yet, it is less clear how socio-demographic characteristics such as the concordance/discordance between proxy–patient gender, age, and education might influence proxy accuracy. Results from the literature are not completely convergent: in some cases, concordance between patient and proxy ratings appears to depend on those factors [65, 66]; in other cases, no significant results have been identified for proxy characteristics [50, 53, 67–71].

Furthermore, such psychological theories predict the precision. It is well known that people adapt to their conditions, and consequently they perceive them differently [72]. This happens also for the conditions of others. One implication of this theory is that the chronicity of a condition will decrease ratings of that condition, especially in ageing. Research on the attitudes and beliefs showed a discrepancy between self- and external ratings because people are inherently biased in their self-ratings, and also towards others [56]. Also, attitudes, beliefs, and stereotypes towards the patient’s health condition or aging itself affect the role of the proxy because self-schemas (e.g. complex implicit internal theories about which aspects of one’s life and of others are stable, and which are subject to change) reorganize memories of events and experiences in order to accommodate beliefs [73]. In such cases, proxy tend to downplay experiences, disinclining to attribute negative symptoms to the patient when there are pertinent implications for himself. For instance, caregivers may consider themselves as a good caretaker, and this belief may be threatened by the patient’s health condition. On the other hand, a caregiver who believes that being a caregiver compromises his/her life and involves failures in various areas of its existence because of the sacrifices he or she makes as a caregiver, might evaluate the patient symptoms as more serious than they really are, supporting this self-concept [56].

Moreover, an understudied aspect of proxy questionnaires that could increase the inaccuracy of responses is the construction of items for proxy rating. It is broadly agreed that an item represents a stimulus and prescribes a response, but it is also important to understand how proxies respond to items [74]. In some cases, notwithstanding conceptual consistency among items—that is, items stem from a certain theory of measurement and their characteristics complement that theoretical framework—it can be hard for a proxy to know how to respond, when items are not about overtly manifested behaviours but rather about the patient’s covert perspective as manifested in thinking, or retrieved memories, or behaviours that the patient mainly performs alone. For instance, this is the case with some questions used in informant interviews to assess dementia outcomes in older people (e.g. “Does the patient have more trouble remembering things that have happened recently than she used to?”). In fact, research on inter-rater reliability has shown that proxy responses were generally more precise and less systematically biased when evaluating more objective domains—such as reporting patients’ symptoms and specific events [54, 75, 76]. Finally, items employed in self-reported measures are often used unchanged in proxy ratings, without taking the responder’s point of view into consideration.

For one, the motivation of a proxy respondent may differ from that of a target respondent, leading to differences in effort, and potential errors in responses provided by the proxy respondent [77]. Proxy respondents may also base their answer on their own behaviours and attitudes and guess instead of using a recall strategy [77, 78]. Respondents’ perspectives also vary depending on whether they are providing a self versus proxy response, and this can influence response quality and error rates. Proxy responses are not based on self-knowledge or first-hand knowledge of events, but rather on the perception of events that happened to someone else, which may be less accurate, detailed, or salient [79, 80].

Finally, another psychological construct that can be considered when proxy responses are analysed is their level of confidence. This concept has recently been deemed important because of its predictive validity for responses [81]. There are two methodologies that measure confidence: a self-report measure, and a measure described as “online.” The online measure is a post-task question, which asks the respondent to rate how confident they are that their answer was correct [82]. The measurement of online confidence developed from early research on decision-making, which employed the use of accuracy ratings in relation to items on cognitive tests [82]. These measures have been described as “online” because they relate to a just completed task and involve a metacognitive judgment of accuracy. Cognitive, personal, and motivational factors influence the level of confidence such as gender with mixed findings [83], personality factors [84] as well as openness and conscientiousness, trait factors as optimism [85], and finally self-concept and self-efficacy as a cognitive representation of one’s own abilities and expectations about successful task completion, respectively [84].

## The present study

What if a direct comparison between proxy and patient responses is not possible? As stated by Elliot and colleagues [54], we should treat proxy responses to subjective ratings cautiously. Our study was developed to provide clinically useful answer to this question. We were also motivated by the need to bring more attention to the important topic of proxy reliability, a topic that has been a bit neglected by recent literature yet remains essential and compelling from a clinical point of view.

The Introduction highlights some sources of variation that must be considered in the evaluation of proxy responses. If it is not possible to compare the responses of the proxy with those of the patient or this is not feasible, for example because the latter is affected by a neurocognitive disease or brain injury, it might be useful to find strategies to evaluate proxy reliability per se*,* with no need for a direct comparison with the patient’s responses.

The smartest strategy would be to evaluate the proxy’s level of knowledge of the patient (e.g. time living together, frequency of contact, etc.). However, as reported in the Introduction, several types of behaviour are hard to evaluate because, for example, they relate to internal states, and proxies’ tendency to respond inaccurately to this kind of question (i.e. to exhibit response bias) cannot be excluded.

We decided to focus on an older adults’ population, considering evidence from the literature that reports from older individuals and their proxies are not highly related on domains such as quality of life and psychological wellbeing [20, 82] but show relatively good agreement with respect to the patient’s functional status, instrumental activities of daily living, and cognitive status [20, 62, 86, 87].

The general aim of the study was to fine-tune a reliable method for evaluating the accuracy of proxy responses when the patient’s answers cannot be considered fully reliable. As mentioned above, this is the situation that most often leads a clinician to turn to a proxy. Ahasic and colleagues [88] previously explored the reliability of assessments made by carefully chosen proxies, by evaluating agreement between patient and proxy responses when assessing activities of daily living (ADLs). Their research suggests that carefully chosen proxies tend to provide reliable information on patients’ functional status and recommend systematic screening to determine the reliability of proxy responses.

The basic idea here is straightforward since it consists in exploiting the difference in proxy responses to question probing the frequency of the patient’s overt versus covert behaviours. The rationale is that reliable and consistent proxies will show signs of being in trouble or uncomfortable when answering questions about covert behaviours. As showed by Evans and colleagues [89], the uncertainty to respond differs according to the nature of the question being asked, generating a feeling of inadequacy in the proxy. Operationally, this difficulty should be manifested as more frequently replying “I don’t know” to for covert than overt questions. To demonstrate the validity of this effect, our study had the following specific aims:

1. To develop and then test the psychometric properties of a set of new items, examining their content validity. For this purpose, we first collected an initial sample of 12 items, reflecting covert and overt behaviours inspired by both indoor and outdoor activities of basic/instrumental daily living. We then asked five experts to provide a qualitative assessment of how well these items elicited information on overt or covert behaviours. Next, we performed exploratory and confirmatory factor analyses (EFA and CFA, respectively) to test the factorial validity of the questionnaire. The final set included 10 items (Study I);
2. To demonstrate the criterium validity of the questionnaire, we employed a sample of healthy older adults and their proxies in a first sub-study, to ascertain a) how response precision, namely the degree to which the proxy’s response agreed with the patient’s response, was lower in the case of items characterizing covert as compared to overt behaviours, b) that the items about covert behaviours generated more “I don’t know” answers than those that represented overt behaviours, and that the level of the proxy’s knowledge of the patient (operationalized here as being or not being a cohabitant) affected the number of “I don’t know” responses. Finally, c) we conducted a replication study (second sub-study) to compare the previous results regarding “I don’t know” answers generated by covert behaviours, using a sample of students who answered questions about their parents. In this case, the level of knowledge students had of their parents was measured by directly asking students to identify the parent they felt they knew best (Study II).

At this point, we asked how the questionnaire could best serve clinicians noting the need for a method that could identify reliable proxies even when direct comparison with patients’ responses could not be made due to the latter’s incapacity. To do this, we decided to validate the effectiveness of our reasoning using online response confidence levels. So, we administered the 10 items questionnaire to a sample of healthy older adults and their proxies, asking the proxy to also report its confidence in the responses (Study III). We expected that reliable proxies would exhibit differences in confidence for overt versus covert behaviours.

## Study I–content and factorial validity

### Content analysis

The first aim of Study I was to assess the content validity of a set of items designed to probe another person’s overt and covert behaviours. To meet the need for a reliable and valid instrument for assessing both indoor and outdoor covert and overt behaviours, two Authors (AL, AB) developed a pool of 20 test items, in order to start from a streamlined and easy to use tool. After conducting an extensive review of the literature on overt and covert behaviours, identifying the constructs in the theoretical context of interest, they selected those that best represented the constructs and met the guidelines of Clark and Watson [90, 91] for item-writing leading to 12 items. The items addressed common problems encountered when shopping, going for a walk, driving, preparing meals, taking medications, and handling money [92, 93], items compatible with the IADLs, complex activities related to the ability to live independently in the community. Including the basic ADLs, skills required to manage one’s basic physical needs, could have meant intercepting people in a more advanced clinical condition. The 12 original items together with the response modality are shown in Table 1.

**Table 1:** Item questionnaire, overt and covert behaviour (O/C) and Fleiss' Kappa agreement results of five raters

Item		O/C	Fleiss’ kappa
Q1	Going through a red light as a driver or pedestrian	O	1.00
Q2	Responsibly dispensing own medication or pills	O	1.00
Q3	Switching to another lane, or suggesting a switch because the lane was going to be blocked	O	1.00
Q4	Moving forward without respecting the queue (e.g., at the supermarket, post office, bank)	O	1.00
Q5	Confusing tasks, for example, taking the juice instead of milk box, because lost in thought	C	1.00
Q6	Forgetting that they had already just used an ingredient while preparing a meal	C	1.00
Q7	Turning the corner to find themselves next to another person they had not seen, risking bumping into him/her (e.g., while shopping)	O	1.00
Q8	Getting into the bathtub or shower when no one is home	C	1.00
Q9	Driving a vehicle after drinking	O	1.00
Q10	Realizing/noticing that they paid a bill without checking the change, because lost in thought	C	1.00
Q11	Walking without paying attention to the road	C	1.00
Q12	Forgetting to switch the stove off, because distracted	C	1.00

#### Method

A panel of five expert raters, psychologists and psychotherapists expert in psychology of ageing (2 females, age M ± SD: 40.80 ± 7.46, education M ± SD: 21.20 ± 0.40), were asked to judge the 12 items as representative of overt or covert daily living behaviours. Expert evaluation of the concordance was performed through Fleiss’ kappa index [94].

#### Results

Fleiss’ kappa returned a high coefficient of concordance well above chance. As reported in Table 1, raters achieved perfect agreement. They identified six items covering overt behaviours and six involving overt behaviours.

### Exploratory factors analysis

The second aim of Study I was to examine the psychometric properties of the pool of 12 items by testing its dimensionality through an EFA.

#### Method

##### Participants

One hundred and sixty-two young adults (93 females, between 19 and 35 years of age; age M ± SD: 23.77 ± 3.08, education M ± SD: 16.52 ± 1.33) took part in the study. All the participants were enrolled from January to February 2021. They were university students who responded to an advertisement and participated without compensation. All participants were blind to the hypothesis of the study and provided informed consent. Participation was anonymous and voluntary. The inclusion criterium for young adults was academic performance considered as a measure of cognitive efficacy [95–98]. Participants had high/adequate academic achievement measured as the number of exams per years (inclusion cut-off: five or more exams; maximum number of exams per year: seven). The choice to enrol young adults was due to the notion of an early onset of cognitive decline [99], lowering the age of possible proxies. The local Ethical Committee of the Institution approved the study protocol (ET-21-01). Following Hair and colleagues [100], an acceptable sample size for EFA, as well as for CFA, would include observations equal to 5 times the number of observed variables (i.e. items), while a more acceptable ratio would be 10 times the number of observed variables. In the present study, there were 162 observations for 12 observed variables.

##### Materials and procedure

The 12 items, based on a 5-point Likert-type scale, that provides respondents with a manageable range of options to choose from never (1) to always (5), were shown to the participants, together with a short general anamnesis requiring demographic information. The entire procedure was explained to the participants beforehand. Participants were assessed individually in a well-lit and quiet room without disturbances. Data were collected in a single session. The whole assessment lasted no more than 20 min.

##### Statistical analysis

The data were analysed using the R software packages psych [101], MVN [102], and lavaan [103]. The assumptions of normality, linearity, homogeneity, and homoscedasticity were checked to identify any violations. Measures of reliability and validity were obtained by measuring internal consistency (Cronbach’s α) and performing EFA according to Arifin’s guidelines [104].

##### Results

Data were not normally distributed at the multivariate level. The subsequent principal axis factoring (PAF) extraction method was applied to deal with this non-normality. To check the suitability of the data for analysis, we applied the Kaiser–Meyer–Olkin (KMO) Measure of Sampling Adequacy (MSA) and found KMO was equal to 0.80, that is, meritorious [105]. Bartlett’s test of sphericity confirmed significant correlations between the items (*χ*^2^ = 668.641; *p* < 0.001). Cronbach’s alpha was used to examine the internal consistency of items. Any value above 0.7 is usually considered to indicate acceptable reliability for any given scale [106]. Here, the Cronbach’s alpha of the 12 items composing the scale was 0.82. A parallel analysis, performed to determine the number of relevant factors, suggested two factors.

We next ran EFA. As stated above, the data were not normally distributed. For this reason, we chose PAF as the extraction method, because it does not assume normality of data [107] and used the recommended rotation method, Oblimin [108]. The quality of items was then assessed; those that did not load adequately were not well-correlated with their factors. The starting point was to extract the number of factors equal to those suggested by the scree-plot (i.e. two). Two items did not load adequately, that is, above 0.30 [100]. To develop a slender tool, we chose the best five descriptors for each component with adequate communalities, factor loadings, and low item complexity [109–112] according to Table 2, excluding two items with factor loadings below 0.5 (factor loadings “Practically Significant”, [100]). These items were (Q8) Getting into the bathtub or shower when no one is home, and (Q9) Driving a vehicle after drinking.

**Table 2:** Exploratory factor analysis, two-factor model, 12 items (*N* = 162) including factor loadings, communality, and item complexity

Item	Factor 1	Factor 2	Communality	Item complexity
Q1	− 0.06	**0.579**	0.305	1.02
Q2	0.107	**0.665**	0.521	1.05
Q3	0.141	**0.622**	0.491	1.1
Q4	− 0.06	**0.739**	0.507	1.01
Q5	**0.51**	0.047	0.244	1.02
Q6	**0.618**	0.038	0.406	1.01
Q7	− 0.111	**0.581**	0.288	1.07
Q8	0.366	0.078	0.167	1.09
Q9	0.026	0.482	0.246	1.01
Q10	**0.922**	− 0.006	0.845	1
Q11	**0.743**	− 0.021	0.537	1
Q12	**0.548**	0.032	0.318	1.01

Bold values represent factor loadings greater than 0.3

The final set comprised 10 items. The two factors were: Covert Behaviours, (Factor 1), and Overt Behaviours (Factor 2). Next, we checked the internal consistency reliability of the factors extracted in the EFA with Cronbach’s alpha. We ascertained the reliability of each factor separately by including the selected items per factor. The Cronbach alphas (0.78 for each factor) indicated adequate internal consistency reliability [113, pp. 95–96]. The total scale reliability was 0.82, which is considered good. The two factors seemed to share a remarkable amount of variance (*r* = 0.487), as was expected for two facets of the same construct, that is, common daily activities.

### Confirmatory factor analysis

Two hundred and forty-four participants were employed to validate the previous analysis by performing a CFA.

#### Method

##### Participants

Overall, 244 young adults, 104 females, between 19 and 35 years of age (age M ± SD: 22.41 ± 2.73), took part in the study, enrolled in the same way as for Study II. The mean level of education for the overall sample was 15.72 years (SD = 0.96 years). The inclusion criterium was the same of EFA.

##### Materials and procedure

Setting and materials were the same as in EFA.

##### Statistical analysis

CFA was performed using maximum likelihood (ML) estimation to test the construct validity of the identified EFA structure and the fit of the set of items. The adequacy of fit was assessed using the relative Chi-square (*χ*^2^/df ≤ 2, [114]), comparative fit index (CFI) ≥ 0.95, Tucker–Lewis Index (TLI) ≥ 0.95, root-mean-square error of approximation (RMSEA) ≤ 0.06, and standardized root mean square residual (SRMR) ≤ 0.08 [115].

##### Results

**Fig. 1:**
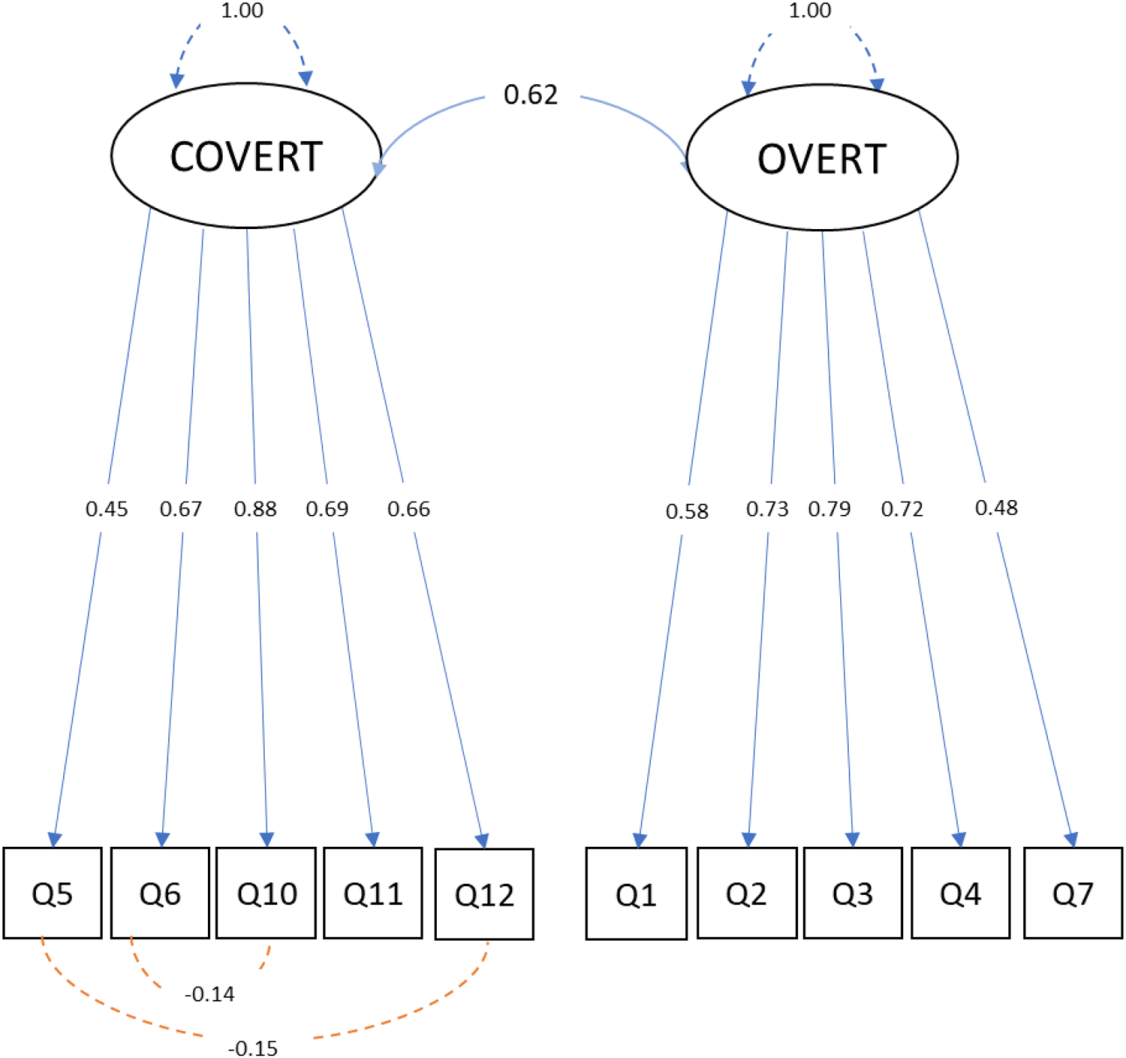
Two-domain confirmatory factor model, including factor correlations

CFA was used to test the construct validity of the identified two-domain factor structure of the set of items (see Fig. 1). As the standard Chi-square test may not be a reliable indicator to model adequacy [116], the relative Chi-square fit index (*χ*^2^/df) was also considered (values less than two have been suggested to represent “good” data-model fit, [117]). The relative Chi-square fit index for this model satisfied the recommended cut-off values (*χ*^2^/df = 1.8). Accepted values were also found for four other “goodness-of-fit” indices (*χ*^2^ = 60.32; *p* ≤ 0.001; CFI = 0.96; TLI = 0.96; RMSEA = 0.06; SRMR = 0.04), suggesting a good fit between the hypothesized model and the observed data [115, 116]. The correlations between factors were also confirmed by CFA. Recurring to the modification indices, the residual co-variances of Q5 and Q12, and Q6 and Q10 were unconstrained to obtain a model with better fit.

##### Discussion

A new questionnaire was developed, including items that describe overt and covert behaviours in activities of daily living. A panel of five experts was employed to assess content validity. Applying EFA to 12 items yielded a two-factor model with 10 items reflecting overt and covert behaviours with good internal consistency. This model was confirmed by CFA, and construct validity was also supported by the correlations between the two factors. The two factors were called overt and covert behaviour, each consisting of five items. The 10 items were used to produce a questionnaire we named the Proxy Reliability Questionnaire (ProRe). This is a classic questionnaire including questions relating to the frequency of behaviours (overt and covert) in a person of whom s/he had direct and in-depth knowledge (like an older adults’ parent or spouse).

### Study II–criterium validity

To pursue the aims of this study, the Proxy Reliability Questionnaire (ProRe) was administered to a sample of healthy older adults and their proxies. Two versions of the questionnaire were employed. The difference between the two versions was that the proxy could provide the answer “I don’t know” in addition to the other answers related to frequency (never, rarely, occasionally, quite often, almost always). It was expected that proxy “I don’t know” answers would appear significantly more often in response to questions regarding covert behaviours.

(A) The occurrence of “I don’t know” answers for overt and covert behaviours

#### Method

##### Participants

First, a power analysis was carried out with G*Power 3.1 [118] to estimate the sample size (*p* level = 0.05; a cautious low effect size = 0.10); and power = 0.80). Results indicated that a sample size of about 90 participants was adequate to warrant an 80% chance of correctly rejecting the null hypothesis. The final sample included ninety-two healthy older adults’ persons (mean age ± sd 71.25 ± 6.66; mean level of education in years ± sd 10.22 ± 4.16), each coupled with a proxy informant. The older adults and proxy informant (mean age ± sd 46.64 ± 17.20; mean level of education in years ± sd 14.03 ± 4.12) were all first-degree relatives (e.g. spouse or son/daughter) who did or did not live together. They were volunteers recruited by word of mouth enrolled in the study from April to May 2021. All participants were blind to the hypothesis of the study and provided informed consent. People with a history of suspected uncompensated systemic/traumatic/psychiatric disease or with a severe vision/hearing loss that could have affected cognition were excluded from the final sample according to the results of a preliminary general anamnesis carried out by three supervised trainees in psychogeriatric assessment. Moreover, global cognitive function was assessed through the General Practitioner Assessment of Cognition test (GPCOG, [119, 120]). The inclusion cut-off was a GPCOG score above 7 (healthy patients mean ± sd 7.89 ± 0.31; proxy informant mean ± sd 8.94 ± 0.22), which had been shown to be the optimal cut-off for discriminating healthy participants from participants with probable cognitive impairment in an Italian sample of older adults [120]. Finally, the Activities of Daily Living and Instrumental Activities of Daily Living (ADL, [121]; IADL [122],) were administered to control for any possible occurrence of functional decline usually associated with dementia (inclusion cut-off higher than 4 for ADL: healthy patients mean ± sd 5.86 ± 0.36; proxy informant mean ± sd 5.90 ± 0.29; higher than 4 for males and 6 for females for IADL: healthy patients mean ± sd 6.97 ± 1.20; proxy informant mean ± sd 7.13 ± 1.10). No one was excluded from the sample.

##### Materials and procedure

Two versions of the ProRe questionnaire were created (see Supplementary materials), one for the healthy patient who answered for himself, and one for the proxy who provided responses about the patient’s behaviours*.* The items were administered in the form of questions (e.g. patient version: How often does it happen that you…?; Proxy version: Did Mr/Mrs …?) on a 5-point Likert-like scale from never (1) to almost always (5). Moreover, proxy informants were given the option to respond “I don’t know” in cases where they had no clear memory of item content or were not completely confident in their answer. All the participants completed the questionnaires in Italian through an online survey platform (Google Forms) under the direct control of the research assistants. The Ethical Committee of the Institution approved the general study protocol (n. ET-21-01), and the whole study was performed following the Helsinki Declaration and its later amendments.

##### Statistical analysis

- To compute the measure of agreement (beyond chance) between the patient and the proxy, we computed weighted K statistics. This is the standard statistical approach to evaluate response precision between patients and external raters [62], an indicator of inter-rater reliability. The k interpretations by Landis and Koch [123] could be applied to guide interpretation of patient/proxy agreement as poor (< 0.20), fair (0.21–0.4), moderate (0.41–0.60), or substantial (0.61–0.80). Moreover, another way to compute the response precision was used. We evaluated the amount and direction of response discrepancy between the patient and the proxy respondent with the discrepancy score (proxy score–patient score). The main idea of this study was to verify the concordance between patient and proxy, comparing two classes of behaviours. The deviations in scores are interpreted purely as discrepancies, so this score can become a useful marker of the gap between the patient and the proxy point of views [56].
- Then, a mixed factor ANOVA was performed with behaviour (two levels: overt and covert), as repeated measure, the status of cohabitants (two levels: cohabitants, not cohabitants) as between factor, and the number of “I don’t know” answers as the dependent variable, using gender and age of participants as covariates.

##### Results

First, we evaluated the discrepancy between proxy and patients’ evaluations. Table 3 presents this data reporting the values of weighted K statistics—the discrepancy score—and the number of “I don’t know” answers. As can be seen from the table, overt behaviours are more easily assessed than covert ones. Accordingly, the gap between the proxy and the patient evaluation shows greater discrepancy for covert compared to overt behaviours. The number of “I don’t know” answers is significantly higher for covert behaviours.

**Table 3:** Response precision between patients and external raters, and number of “I don’t know answers” for each item

N.	Questions	Behaviour	Weighted K	Mean discrepancy score (95% CI)	Number of I don’t know
1	Q1	O	0.68	0.19 (0.07–0.30)	3
2	Q2	O	0.57	0.37 (0.20–0.53)	1
3	Q3	O	0.58	0.29 (0.16–0.41)	2
4	Q4	O	0.46	0.32 (0.19–0.44)	3
5	Q5	C	0.10	0.65 (0.50–0.79)	10
6	Q6	C	0.21	0.49 (0.33–0.64)	10
7	Q7	O	0.69	0.15 (0.70–0.23)	0
8	Q10	C	0.23	0.51 (0.35–0.66)	6
9	Q11	C	0.10	0.58 (0.44–0.71)	6
10	Q12	C	0.22	0.64 (0.47–0.80)	9

**Fig. 2:**
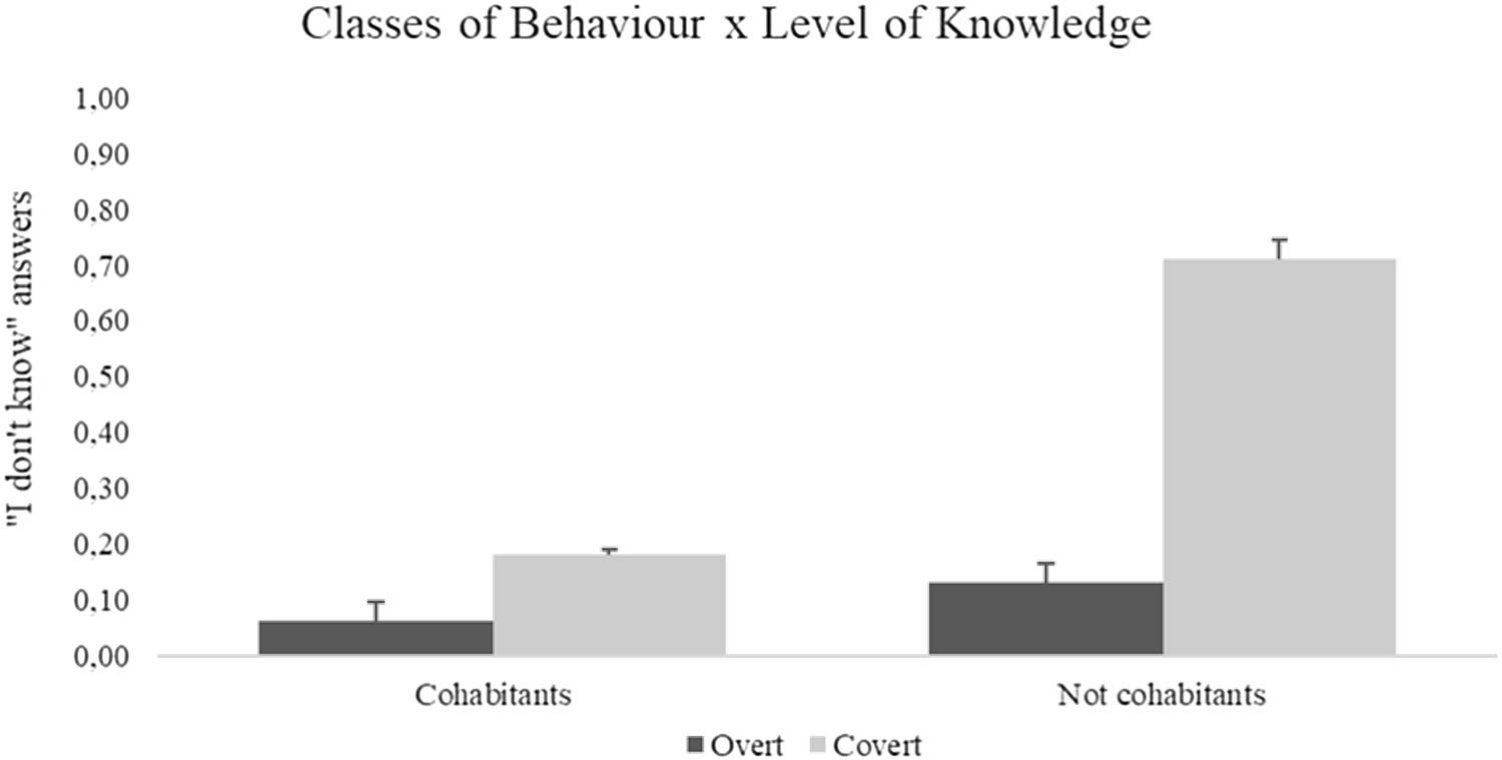
Mean and standard error (95% Confidence Intervals) of “I don’t know” answers for overt (dark grey bars) and covert (light grey bars) behaviours, for cohabitants and not cohabitants

Secondly, we checked whether covert behaviours generated more “I don’t know” answers than those that represented overt behaviours. The results of the mixed factor ANOVA, calculated only on proxy responses, showed the significant main effect of classes of behaviour *F*(1, 85) = 7.52, *p* < 0.01; ${\upeta }_{p}^{2}$= 0.10 (overt: 0.09 ± 0.05; covert: 0.45 ± 0.1), and level of knowledge *F*(1, 85) = 4.25, *p* = 0.04; ${\upeta }_{p}^{2}$= 0.05 (cohabitants: 0.10 ± 0.10; not cohabitants: 0.42 ± 0.10). Moreover, classes of behaviour x level of knowledge, *F*(1, 85) = 6.91, *p* = 0.01; ${\upeta }_{p}^{2}$= 0.07, was also significant. From the inspection of the graph (see Fig. 2), it emerged that the number of “I don’t know” answers was higher for not cohabitants and for covert behaviour. The effects of covariates were not significant.

(B) The occurrence of “I don’t know” answers for overt and covert behaviours: A replication

#### Method

##### Participants

The present section of the study was aimed to replicate and extend the previous results regarding the “I don’t know” answers. Firstly, a power analysis was carried out using G*Power 3.1 [118] to estimate the suitable sample size, using the following parameters: *p* level of 0.05, medium effect size (0.25), and power of 0.80. Results indicated that a sample size of 52 participants was adequate to warrant an 80 per cent chance of correctly rejecting the null hypothesis.

So, to accomplish the third aim of the study (i.e. (c)), a convenience sample of 81 healthy students (40 females), between 18 and 29 years of age (age M ± SD: 20.03 ± 2.47), took part in the study. The mean level of education for the overall sample was 14.38 years (SD = 1.88). All the participants were enrolled from June to July 2021. They were university students who responded to an advertisement and performed the experiment without compensation. All participants were blind to the hypothesis of the study and provided informed consent. Participation was anonymous and voluntary. The inclusion criteria for all participants were: (a) completing the entire survey; (b) cohabiting with the targets of the assessment (i.e. parents); (c) both parents were still in full-time employment; (d) both parents enjoyed good cognitive health. Academic performance was an adequate measure of cognitive efficacy [96, 97, 124, 125]. Young participants had high/adequate academic achievement measured as the number of exams per years (inclusion cut-off five or more exams, maximum number of exams per year: seven). No one was excluded from the sample. Participants freely specified which parent they thought they knew better, their mother or their father, but were nonetheless requested to rate both.

##### Materials and procedure

Participants completed the proxy version of ProRe questionnaire focusing consecutively on behaviours of their parents. Half the sample was asked to first choose and rate the parent who they thought they knew best, and then the parent who they thought they knew less well, vice versa for the other half. Procedures were the same as in the previous sections of the study.

##### Statistical analysis

Preliminary counterbalancing was used to control for any order effects in the choice of the parents (better known, less known) on the outcome variable being measured, namely the number of “I don’t know” responses. For the purposes of the study, a within-subjects ANOVA was performed with classes of behaviour (two levels: overt and covert) and the level of knowledge of the parents (two levels: best known, less known) as repeated measures, and the number of “I don’t know” answers as the dependent variable, using gender and age of participants as covariates.

##### Results

**Fig. 3:**
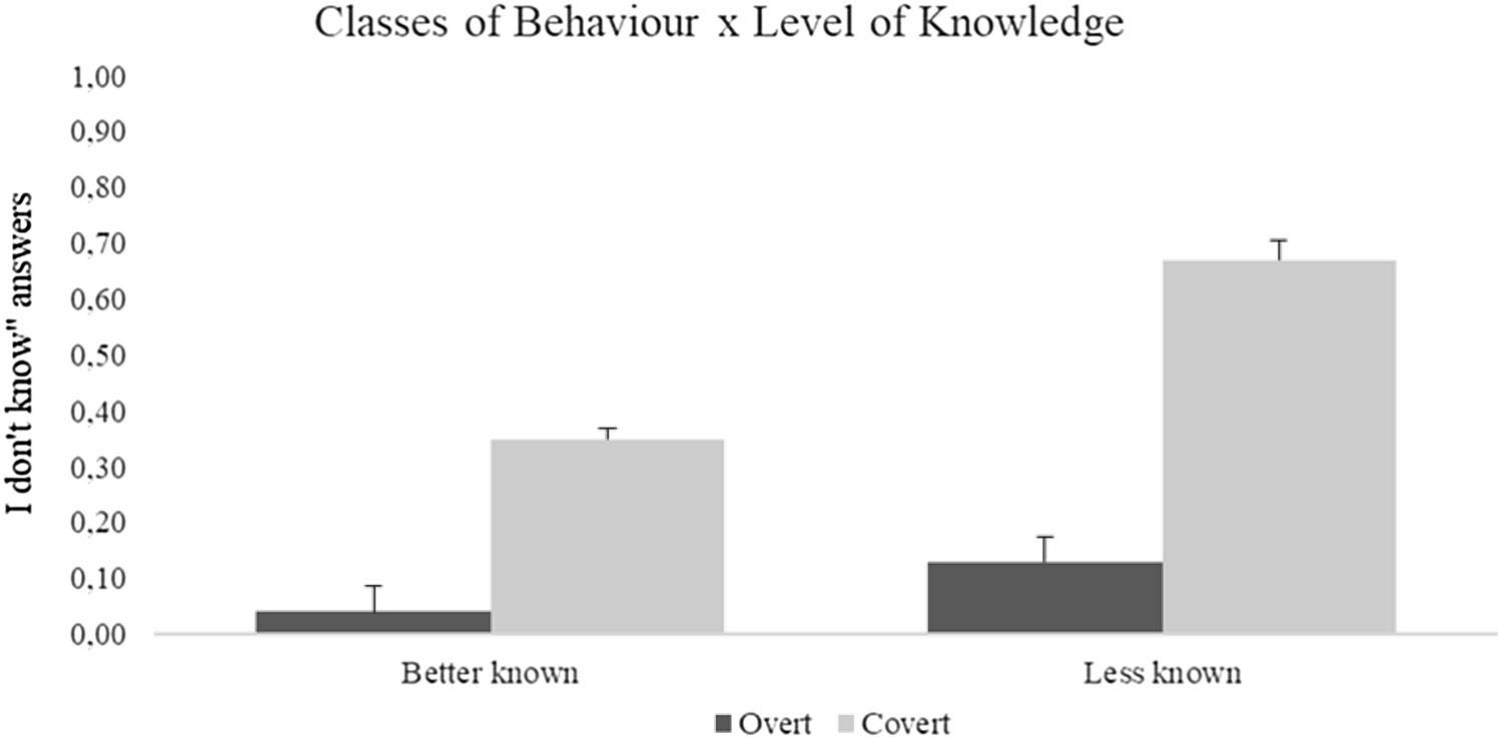
Mean and standard error (95% confidence intervals) of “I don’t know” answers for overt (dark grey bars) and covert (light grey bars) behaviours, for the better known and less known parent

The main effect of classes of behaviour *F*(1, 75) = 11.13, *p* = 0.01; ${\upeta }_{p}^{2}$= 0.12 (overt: 0.19 ± 0.05; covert: 0.40 ± 0.06), and level of knowledge of the parents *F*(1, 75) = 16.53, *p* < 0.001; ${\upeta }_{p}^{2}$= 0.16 (best known: 0.08 ± 0.02; less known: 0.52 ± 0.10) proved to be significant. Moreover, classes of behaviour x level of knowledge, *F*(1, 75) = 4.50, *p* = 0.03; ${\upeta }_{p}^{2}$= 0.05, was also significant. From the inspection of the graph (see Fig. 3), it emerged that the number of “I don’t know” answers was higher for the less known parents and for covert behaviour. The effects of covariate were not significant.

##### Discussion

In order to verify the criterium validity of the Proxy Reliability Questionnaire (ProRe), we employed two different samples: the first was composed of a group of healthy seniors each with his/her proxy (cohabitants or not cohabitants), while the second comprised students who lived with their parents.

It is often unclear if items in proxy reports refer to objective or subjective events. If survey questions are about objective public circumstances or events, a proxy is more likely to be accurate. When the questions are about events or circumstances that are more subjective or private, involving thinking or reasoning processes, there is an increased risk of receiving inaccurate answers [126]. Indeed, many response biases lead respondents to give answers even when it would be correct/appropriate to refuse to answer at all [127]. Moreover, comparing responses of patients and their proxies, the latter were more accurate in predicting patients’ overt behaviours and had difficulty estimating covert behaviours. This result is consistent with the notion that overt behaviours are more easily assessed than covert ones, because they rely on frequent observation of activities of daily living. It is consistent that being faced with covert behaviour in a condition of lower knowledge increase the likelihood of answers “I don’t know”. As reported in the Introduction, this evidence should make the clinician aware that proxies find some questions hard to respond to [74], but it is unlikely that they will reveal this fact spontaneously. Overall, the findings of the present study suggested that (a) the degree to which the proxy’s response agrees with the patient’s response is lower in the case of items characterizing covert compared to overt behaviours (Section A of the study); (b) the items concerning covert behaviours generated significantly more “I don’t know” answers than those that represented overt behaviours (Sections A and B of the study) and finally (c) comparing different levels of patient/proxy exposure (cohabiting or not, Section A of the study; asking the proxy to judge the best known parent, section B of the study) revealed that “I don’t know” answers were significantly more frequent for covert behaviours and, in turn, for less known parents.

Proxy respondents facing questions regarding covert behaviours might base their answer on their own behaviours and attitudes or appeal to their own beliefs instead of recurring to a recall strategy [78, 126]. Also, ageism could influence the answers [128]: stereotypes associated with aging may influence treatment decisions. Moreover, people have low levels of health literacy regarding ageing [129] that can lead to exaggerated negative responses associated with covert behaviour. Researchers and clinicians could reframe attitudes toward aging, through the mitigation of prejudices and increasing knowledge, allaying the impact of this response bias. In these conditions, the respondent is not answering based on first-hand knowledge of actual events, but rather on their perception of events, which may be less accurate, detailed, or salient [79, 80]. Furthermore, it is known that agreement between proxy and self-responses can vary based on the relationship between the proxy and the individual they are reporting on or on behalf of [130–132].

Higher non-response rates for some items by a proxy may indicate either that a proxy respondent does not have sufficient knowledge or that they are not comfortable providing responses to such questions [133]. In the present study, (a) cohabitating (vs. not cohabitating) with the patient, and (b) identifying a cohabitating parent as better known, were associated with reduced “I don’t know” responses. This evidence revealed that proxy respondents’ answers should be considered as accurate as, or more accurate than, responses that would have been given by subjects themselves [134]. The present–concurrent–criterion validity study suggested that a questionnaire that includes questions about both overt and covert behaviours that (those that not directly observable or which refer to mental states) can detect reliable respondents. Such respondents will provide more “I don’t know” answers or rate their confidence lower on covert than on overt questions. As a limitation of this study, it should be noted that the relationship between an older adult and his/her proxy is likely to be much more complex, due to numerous and intricate changes in the older adult's functioning, than student–parent one, and this could affect the answers.

### Study III–confidence as a measure of the reliability of proxy respondents

Study III aimed to overcome a limitation that emerged from the previous two studies, regarding participants’ tendency to provide what they perceived to be desirable responses. This could be considered a sort of social-desirability bias, namely the tendency of survey respondents to answer questions in a manner that will be viewed favourably, instead of admitting that they cannot provide a response. We know that there are four cognitive recall states (available, accessible, generatable, ignorant) that characterized the response bases framework [135]. The ignorant state refers to one in which the requested information is not known, and there is no basis to approximate this information. On the one hand, respondents might avoid explicit “I don’t know” answers, providing substantive answers under a broad range of conditions, that is, both in cases where they have precise answers and are making wild guesses (error of commission). On the other hand, the presence of “I don’t know” answers might encourage less motivated respondents to select them as an “easy out” (error of omission). These possible respondent tendencies must be taken seriously when using proxy responses on behalf of a patient. To remedy this problem, we introduced the use of online confidence evaluation of each response. Moreover, studies have shown that confidence better predicts response accuracy when it is measured after a decision related response [136–139].

Usually, the “I don’t know” responses were not very abundant, indicating that many respondents tried to respond to each question even when it was inappropriate, indicating they suffered from some kind of survey/response bias. The most common online measures come in the form of a numerical confidence rating yoked to individual items in an ability task. Following each item, the respondent is asked to give a confidence rating in response to the question “How confident are you your answer is correct?”.

#### Method

##### Participants

For this study, a power analysis was carried out with G*Power 3.1 [118] to estimate the sample size (*p* level = 0.05; a cautious low effect size = 0.10); and power = 0.80). Results indicated that a sample size of about 90 participants was adequate to warrant an 80% chance of correctly rejecting the null hypothesis. The sample consisted of 90 healthy older adults (mean age ± sd 78.60 ± 6.30; mean level of education in years ± sd 7.76 ± 4.79), each coupled with a proxy informant (mean age ± sd 52.60 ± 8.70; mean level of education in years ± sd 12.23 ± 3.70). Older adults and proxy informant were first-degree relatives (e.g. spouse or child), cohabitants or not and were enrolled in the study from September to November 2022. The inclusion/exclusion criteria were the same as for Study II (GPCOG, healthy patients mean ± sd 8.04 ± 0.66; proxy informant mean ± sd 8.35 ± 0.20; ADL, healthy patients mean ± sd 5.83 ± 0.31; proxy informant mean ± sd 5.87 ± 0.25; IADL: healthy patients mean ± sd 6.98 ± 1.04; proxy informant mean ± sd 7.05 ± 0.96). No one was excluded from the sample.

##### Materials and procedure

The two versions of the ProRe questionnaire were administered, one for the healthy patient who answered for himself, and one for the proxy who provided responses about the patient’s behaviours with the addition of “I don’t know” answers and online confidence ratings (How confident are you your answer is correct?) along a 5-point Likert-like scale from very unsure (1) to very sure (5).

##### Statistical analysis

First of all, in order to verify the concordance between patient and proxy, the discrepancy score was calculated on overt and covert behaviours. Then, a mixed factor ANOVA was performed with classes of behaviour (two levels: overt and covert) as repeated measures, level of knowledge (two levels: cohabitants, not cohabitants) as between factors, and discrepancy as the dependent variable, using gender and age of participants as covariates. Moreover, a mixed factor ANOVA was performed with classes of behaviour (two levels: overt and covert) as repeated measures, level of knowledge (two levels: cohabitants, not cohabitants) as between factors, and confidence as the dependent variable, using gender and age of participants as covariates.

##### Results

We checked whether covert behaviours generated more discrepancy between healthy patients and their proxies than those that represented overt behaviours. The results of the mixed factor ANOVA showed a significant main effect of classes of behaviour *F*(1, 84) = 8.23, *p* < 0.01; ${\upeta }_{p}^{2}$= 0.10 (overt: 0.27 ± 0.05; covert: 0.59 ± 0.05), and level of knowledge *F*(1, 84) = 21.15, *p* < 0.001; ${\upeta }_{p}^{2}$= 0.20 (cohabitants: 0.24 ± 0.07; not cohabitants: 0.62 ± 0.04). The first-order interaction effect was not significant. The effects of covariates were not significant.

Moreover, we investigated differences in confidence between overt and covert behaviours. The results of the mixed factor ANOVA showed the marginal significant main effect of classes of behaviour *F*(1, 84) = 4.04, *p* = 0.04; ${\upeta }_{p}^{2}$= 0.05 (overt: 4.37 ± 0.06; covert: 4.17 ± 0.08), and level of knowledge *F*(1, 84) = 12.00, *p* < 0.001; ${\upeta }_{p}^{2}$= 0.13 (cohabitants: 4.05 ± 0.06; not cohabitants: 0.49 ± 0.09). The first-order interaction effect was not significant. The effects of covariates were not significant.

##### Discussion

Generally, people feel obliged to provide answers and comply with requests made by researchers [140]. It may also happen that a person gives an answer even when his/her confidence in that answer is low. To overcome this distortive effect, we introduced online confidence ratings, to investigate if a difference in confidence between overt and covert behaviours was present.

The starting point of the present study was to replicate the results obtained in the previous study concerning the accuracy with which a proxy responds to answers regarding overt and covert behaviours. Using the discrepancy score (proxy score–patient score), it emerged that covert behaviours were more difficult to identify than overt behaviours and a higher level of knowledge of the patient, as well as living together, generated a lower level of discrepancy.

The use of the online confidence ratings made it possible to solve the problems described above with the “I don’t know” answer option. The online confidence ratings can be considered marginally significant indicators of proxy accuracy but still promising. The slight difference between overt and covert behaviours represented an important starting point for continuing to use confidence ratings as a measure of proxy reliability.

Probably, as a limitation of this study, the item wording should be improved in order to tap into information reflecting proxy confidence in evaluating patient behaviours. Moreover, the proxy’s level of knowledge of the patient was found significant even when the confidence rating measure was taken into account.

## General conclusion

In some clinical situations, patient-reported data are either unavailable or missing. Employing proxy respondents such as family members is a commonly suggested strategy to address this issue [141, 142].

The present study aims to encourage clinicians and researchers to further research the importance of the relationship between the person under evaluation and the proxy respondent, as well as on the content of items and the type of information these items aim to detect. Coming from the same family (cohabiting or not) with a patient who faces with some kind of barriers to providing their own answers to health professionals does not guarantee accurate reports on that person's characteristics, habits, behaviours, goals, preferences. The core aim of this study was to analyse two classes of patient behaviours that are commonly evaluated—overt and covert behaviours—and a source of variation in the evaluation of proxy responses, namely the patient–proxy relationship, which may affect the reliability of the proxy. In our view, the systematic comparison of evaluations of a small number of situations regarding overt and covert patient behaviours could allow the clinician to evaluate the reliability of the proxy. This vision is in line with the European Medicines Agency (EMA) which underlines that proxy reports should “include only events or behaviours that can be observed” [143, pp. 11–12]. Moreover, only confidence ratings may help to address a possible response bias generated by “I don’t know” answers.

We believe that reflecting on the issue of the reliability of proxy informant and the construction of items for proxy rating is of paramount importance given the goal of obtaining accurate assessments of the patient [144]. This work might represent a starting point and a valuable source of knowledge for those researchers want to contribute further to the topic, and it has limitations. We used convenience sampling to recruit participants, which may limit the generalizability of the findings to other populations. Additionally, the study did not measure divergent or convergent validity, which may limit the accuracy of the results. It is also important to note that the study did not account for the potential influence of caregiver burden or other individual characteristics that were not assessed or controlled for.

More research is clearly necessary to improve the ProRe questionnaire and the online confidence ratings. Future directions should be devoted to (a) the possibility of jointly use the answer “I don’t know” and the online confidence rating for the purpose of identifying the reliability of the proxy answers in a two-step model, and (b) the appropriateness and relevance to clinical practice of the present research [145]. External validity is another critical focus when applying study results to specific practices and population contexts [146], in order to make study outcomes directly relevant and informative to clinical practice. The next step could be the creation of a structured clinical interview including probing questions designed to reveal if responses are based on observed data, stereotypes, prejudices, or beliefs, allowing the clinician to promptly decide whether the answers provided are reliable or if, instead, they should obtain a second opinion from another relative or to start a more expensive session of direct observation by a trained caregiver in order to ensure they can compile a rigorous and reliable sketch of the patient's clinical condition.

### Supplementary Information

Below is the link to the electronic supplementary material.

Supplementary file1 (DOCX 15 KB)

## Data Availability

The datasets generated during and/or analysed during the current study are not publicly available due to policy of the funder but are available from the corresponding author on reasonable request.
