# Supplementary material for: Measuring the reliability of proxy respondents in behavioural assessments: an open question
Source: Aging Clin Exp Res. 2023 Aug 4;35(10):2173–90. doi: 10.1007/s40520-023-02501-z (PMC10520105; doi:10.1007/s40520-023-02501-z)
Supplement: Supplementary file 1 — Supplementary file1 (DOCX 15 KB) [file 40520_2023_2501_MOESM1_ESM.docx]

In the present supplementary material (S1.) we have reported the Proxy Reliability Questionnaire (ProRe) (proxy version and patient version), used in this work for studying proxy reliability.

**ProRe**

**Proxy Version**

*Instruction*

These ten questions refer to the behaviour of Mr/Mrs _____________ These activities may have occurred today as well as in the past. Please, take your time to consider each question carefully, then respond on the scale provided.

**Never (1), Rarely (2), Occasionally (3), Quite often (4), Almost always (5), I don’t know (6)**

If you have no clear memory of the described action or if you are not completely confident in your answer, you can freely choose the “I don’t know” alternative. It's completely acceptable to not know the answer to some questions.

How often does it happen that Mr./Mrs. _________________...

| Item | Activities | # |
| --- | --- | --- |
| Q1 | … go through the red light, as a driver or pedestrian? |  |
| Q2 | … take responsibility for dispensing their own medication or pills? |  |
| Q3 | … switch to another lane, or suggest doing so because the lane was going to be blocked? |  |
| Q4 | … move forward without respecting the queue (e.g., at the supermarket, post office, bank)? |  |
| Q5 | … confuse tasks, for example, taking the tetra pack juice instead of the milk, because lost in thought? |  |
| Q6 | … forget having already just used an ingredient to prepare a meal? |  |
| Q7 | … turn the corner finding himself next to another person they had not seen, thereby running the risk of bumping into him/her (e.g., while doing shopping)? |  |
| Q10 | … realize/notice they had paid without checking the change, because lost in thought? |  |
| Q11 | … walk without paying attention to the road? |  |
| Q12 | … forget to switch the stove off, because distracted? |  |

**ProRe**

**Patient Version**

*Instruction*

These ten questions refer to your behaviour. These activities may have occurred today as well as in the past. Please, take your time to consider each question carefully, then respond on the scale provided.

**Never (1), Rarely (2), Occasionally (3), Quite often (4), Almost always (5)**

How often does it happen that you…

| Item | Activities | # |
| --- | --- | --- |
| Q1 | … go through the red light, as a driver or pedestrian? |  |
| Q2 | … take responsibility for dispensing their own medication or pills? |  |
| Q3 | … switch to another lane, or suggest doing so because the lane was going to be blocked? |  |
| Q4 | … move forward without respecting the queue (e.g., at the supermarket, post office, bank)? |  |
| Q5 | … confuse tasks, for example, taking the tetra-pak of the juice instead of the milk, because lost in thought? |  |
| Q6 | … forget having already just used an ingredient to prepare a meal? |  |
| Q7 | … turn the corner finding himself next to another person they had not seen, thereby running the risk of bumping into him/her (e.g., while doing shopping)? |  |
| Q10 | … realize/notice they had paid without checking the change, because lost in thought? |  |
| Q11 | … walk without paying attention to the road? |  |
| Q12 | … forget to switch the stove off, because distracted? |  |
